# Phenotype reversion as “natural gene therapy” in Fanconi anemia by a gene conversion event

**DOI:** 10.3389/fgene.2023.1240758

**Published:** 2023-09-18

**Authors:** Ilaria Persico, Ilaria Fiscarelli, Alessandra Pelle, Michela Faleschini, Barbara Pasini, Anna Savoia, Roberta Bottega

**Affiliations:** ^1^ Genomic Instability DNA Repair Syndromes Group, Joint Research Unit in Genomic Medicine UAB-IR Sant Pau, Sant Pau Biomedical Research Institute (IIB Sant Pau), Barcelona, Spain; ^2^ Dipartimento di Scienze Mediche, Università degli Studi di Torino, Torino, Italy; ^3^ SC Genetica Medica U, AOU Città della Salute e della Scienza di Torino, Torino, Italy; ^4^ Institute for Maternal and Child Health—IRCCS “Burlo Garofolo”, Trieste, Italy; ^5^ Department of Engineering for Innovation Medicine, University of Verona, Verona, Italy

**Keywords:** Fanconi anemia, gene conversion, mosaicism, natural gene therapy, phenotype reversion

## Abstract

Somatic mosaicism appears as a recurrent phenomenon among patients suffering from Fanconi anemia (FA), but its direct prognostic significance mostly remains an open question. The clinical picture of FA mosaic subjects could indeed vary from just mild features to severe hematologic failure. Here, we illustrate the case of a proband whose FA familiarity, modest signs (absence of hematological anomalies and fertility issues), and chromosome fragility test transition to negative overtime were suggestive of somatic mosaicism. In line with this hypothesis, genetic testing on patient’s peripheral blood and buccal swab reported the presence of the only *FANCA* paternal variant (FANCA:c.2638C>T, p. Arg880*) and of both parental alleles (the additional FANCA:c.3164G>A, p. Arg1055Gln), respectively. Moreover, the SNP analysis performed on the same biological specimens allowed us to attribute the proband’s mosaicism status to a possible gene conversion mechanism. Our case clearly depicts the positive association between somatic mosaicism and the proband's favorable clinical course due to the occurrence of the reversion event at the hematopoietic stem cell level. Since this condition concerns only a limited subgroup of FA individuals, the accurate evaluation of the origin and extent of clonality would be key to steer clinicians toward the most appropriate therapeutic decision for their FA mosaic patients.

## Introduction

Fanconi anemia (FA) is a rare genetic condition attributable to variants in over 20 protein-coding genes of the FA/BRCA pathway, which preserves genome stability via the resolution of interstrand crosslinks (ICLs) (Kottemann and Smogorzewska, 2013; Bogliolo and Surrallés, 2015). Upon clastogen exposure (e.g., mitomycin C, MMC, and diepoxybutane, DEB), FA cells indeed manifest a typical chromosomal breakage increase and unique multiradial figures, both linchpins of FA first-line diagnostic tests (i.e., the DEB test) (Auerbach, 1988; Auerbach, 2003).

FA genetic instability accounts not only for probands’ main clinical features, such as bone marrow failure (BMF) and augmented hematological and solid tumor hazards (Kutler et al., 2003; Alter et al., 2018), but also for the substantial incidence (∼30%) of mosaic cases among them (Soulier et al., 2005; Ramírez et al., 2021). As a result of blood’s hierarchical nature (Nicoletti et al., 2020), somatic mosaicism springs from reversion or compensatory events in hematopoietic stem/progenitor cells (HSPCs). All these mechanisms hold the potential to restore a wild-type (WT) allele within daughter cells, thereby promoting the correction of recessive genetic syndromes in compound heterozygotes (Nicoletti et al., 2020). Herein, we describe an FA patient characterized by complete loss of one *FANCA* mutant allele at least in the peripheral blood DNA, exhibiting phenotypic reversion at the hematopoietic level.

## Materials and methods

### Patient

A 34-year-old patient was presented to genetic counseling for reproductive issues because their sister died in childhood. The patient had post-axial polydactyly in the right hand and congenital unilateral kidney agenesis without any hematological alterations, cardiac defects, hearing impairment, and ocular anomalies. From their records, the DEB test performed at the age of 7 provided a doubtful positive result at the lower limit. The DEB test repeated on peripheral blood at the age of 34 was negative. Informed consent was obtained for genetic testing, which was conducted in accordance with the Declaration of Helsinki.

### Mutation screening

Genomic DNA was extracted from the patient’s peripheral blood and oral swab. FA genes were analyzed using the Ion PGM system for next-generation sequencing (Life Technologies, Carlsbad, CA), as described in De Rocco et al. (2014).

For Sanger sequencing, PCR was carried out using the KAPA2G Fast HotStart ReadyMix (Kapa Biosystems, Wilmington, MA). PCR products were purified using ExoSAP-IT (Applied Biosystems, Foster City, CA) and sequenced using the ABI PRISM sequencer (Applied Biosystems, Foster City, CA). Nucleotide numbering reflects *FANCA* cDNA with +1 corresponding to the A of the ATG translation initiation codon in the reference sequence (RefSeq NM_000135). Variants identified were searched in the following annotation databases: the Single Nucleotide Polymorphism Database (dbSNP; http://www.hgmd.cf.ac.uk/ac/index.php), Genome Aggregation Database (gnomAD; https://gnomad.broadinstitute.org), Human Gene Mutation Database (HGMD; http://www.hgmd.cf.ac.uk/ac/index.php), and Fanconi Anemia Mutation Database (https://www2.rockefeller.edu/fanconi/).

## Results

### Clinical features of the patient

The patient (II-2) was born with post-axial polydactyly of the right hand, and right kidney agenesis and a history of growth retardation during infancy were reported. II-2 was referred twice (at 7 and 34 years of age) to the Medical Genetics Unit of the University Hospital in Turin due to the clinical diagnosis of FA in the sister (1 year older) (II-1) who developed acute leukemia shortly after the diagnosis of medullary aplasia.

The DEB test performed at the age of 7 revealed chromosomal instability in two different assays: 40%–42% of unstable cells and 0.72–0.94 chromosomal breaks per cells were observed. Blood count at the age of 15 was found to be normal (white cells 6.68 × 10^9^/L, normal leukocyte formula, red cells 4.78 × 10^12^/L, and platelets 230 × 10^9^/L).

At the age of 34, DEB tests on peripheral blood resulted negative for chromosomal breaks, and blood count was in the normal range (white cells: 5.86 × 109/L, normal leukocyte formula, red cells: 4.66 × 1012/L, platelets: 217 × 109/L, and LDH: 165 UI/L). The patient presented hypovitaminosis D, normal thyroid, and renal function. FSH levels were increased (26.8 U/L), with LH and testosterone within the normal range; the sperm count was reduced with a total number of sperm cells lower than 2.000.000/mL. At clinical evaluation, the patient’s height was 165 cm, with a weight of 63 kg; no skin anomalies were identified, and neither hearing nor visual impairments were referred.

### Mutation screening

**FIGURE 1:**
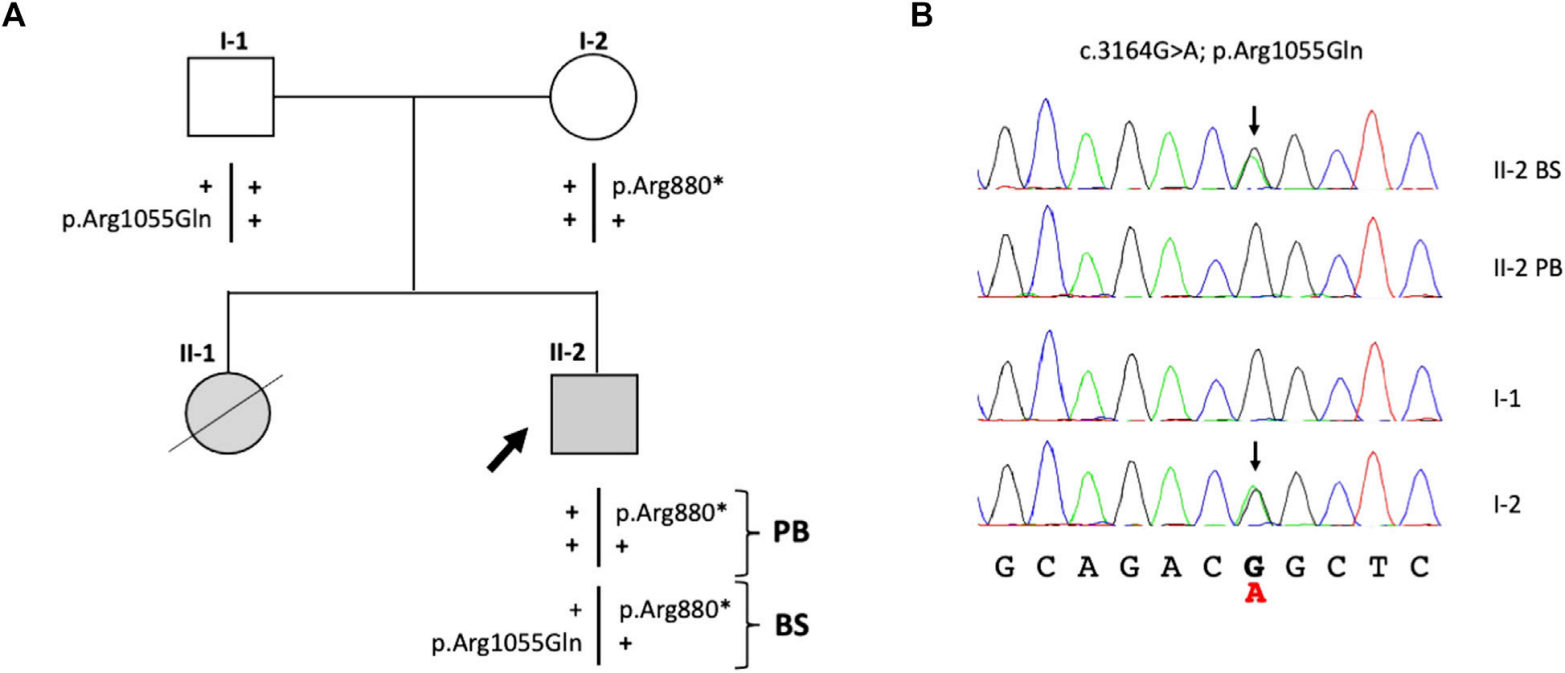
Identification of variants in the *FANCA* gene. **(A)** Patient’s familial pedigree: the black arrow shows the proband (II-2), and plus symbols (+) represent the wild-type alleles. **(B)** Electropherograms of the family members’ variants confirmed by Sanger sequencing. Black arrows indicate FANCA:c.3164G>A substitutions in the heterozygous status found in the mother (I-2) and proband’s epithelial cells. PB, peripheral blood; BS, buccal swab.

Targeted next-generation sequencing (t-NGS) of patient’s (II-2) DNA from PB revealed single-nucleotide heterozygous substitutions (FANCA:c.2638C>T) in exon 28 of the *FANCA* gene, leading to a premature stop codon (p.Arg880*), which was confirmed in the father (I-1) by Sanger sequencing (Figure 1). In the hypothesis of mosaicism, DNA extracted from patient’s buccal swab was analyzed, revealing a compound heterozygous genotype at the *FANCA* gene with the additional FANCA:c.3164G>A substitution (p.Arg1055Gln) inherited from the mother (Figure 1). Both variants, previously reported in the sister (De Rocco et al., 2014), were rare with an allele frequency of 0.003% in GnomAD and were classified as pathogenic in the Fanconi Anemia Mutation Database.

### Mechanism of reversion

**FIGURE 2:**
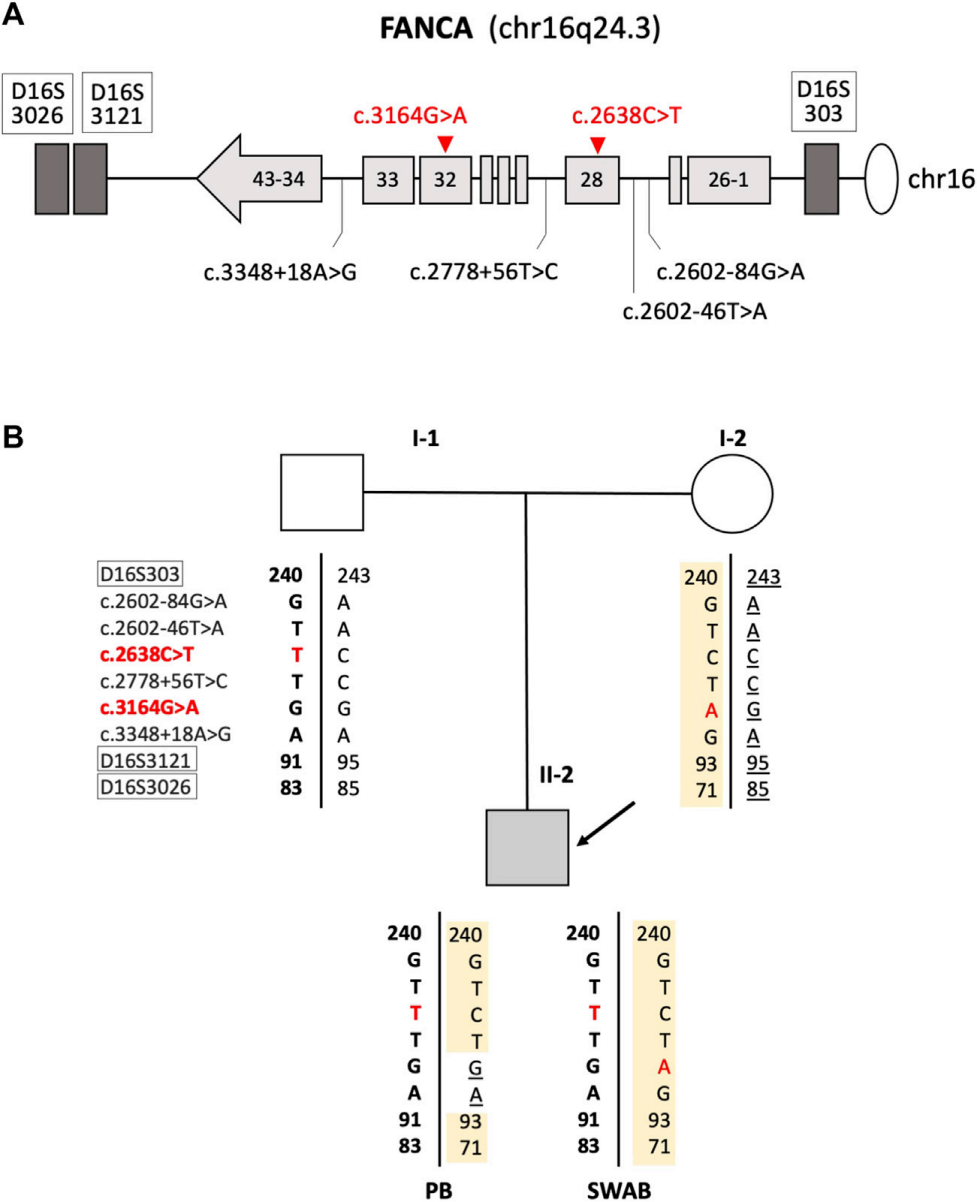
Mechanism of gene conversion: **(A)** schematic representation of the *FANCA* gene with the localization of polymorphic and mutated loci. Mutations found in the patient are reported in red. Microsatellites are indicated inside white boxes. Light gray boxes schematize *FANCA* exons. **(B)** Family’s genotypes at nine loci were within or in close proximity of the FANCA gene.

To study in depth the mechanism responsible for the absence of the FANCA:c.3164G>A substitution in the peripheral blood, we analyzed approximately 2 and 3 kb upstream and downstream, respectively, of the FANCA:c.3164G>A mutation for the purpose of identifying any heterozygote SNP. The only informative SNP was identified in intron 33 (FANCA:c.3348 + 18A>G, rs1800347). It was in homozygous and heterozygous status in DNA from the peripheral blood and oral swab, respectively, suggesting that a gene conversion event was likely to be the underlying mechanism responsible for the mosaic condition observed in the proband (Figure 2).

## Discussion

Hematopoietic mosaicism has been defined as a “natural gene therapy” process and has recently been rated as a good prognostic factor in FA (Ramírez et al., 2021; Nicoletti et al., 2020). Accordingly, our work emphasizes the beneficial effect of the loss of one *FANCA* mutant allele, resulting in the proband (II-2)’s HSPC phenotypic reversion and minor FA clinical features.

The shift of II-2’s DEB test from “mildly positive” in childhood to “negative” in adulthood, together with the patient's family history (dead 8-year-old sister with FA diagnosis) and fertility issues, led us to formulate a clinical suspicion of FA somatic mosaicism. Consistent with this hypothesis, routine genetic analysis on blood DNA demonstrated the presence of the sole paternal *FANCA* allele (FANCA:c.2638C>T, p. Arg880*), while both parental causative variants (the maternal FANCA:c.3164G>A, p. Arg1055Gln) were identified after buccal swab testing. Moreover, the detection of the FANCA:c.3348 + 18A>G SNP in the homozygous and heterozygous status in proband’s PB and oral swab DNA, respectively, allowed us to explain II-2’s mosaicism as the outcome of a gene conversion event. This phenomenon, together with an intragenic crossover and back mutations, is a reversion mechanism that stands out for the unilateral conveyance of the genetic material via homologous recombination between non-allelic or interallelic regions with a high sequence similarity (at least >92%), possibly resulting in the transfer of genetic information from a functional donor sequence to a mutant acceptor sequence (Chen et al., 2007).

Regarding II-2’s clinical course, the bone marrow reversion event explained the complete absence of hematological anomalies observed in our patient since the age of 15, and the presence of the clinical signs of FA (polydactyly, renal agenesis, growth delay, and infertility) is in line with the resultant somatic mosaicism.

Accordingly, recent clinical pictures of blood cell count normalization suggest that FA mosaic patients with a sufficient reversion degree accompanied by the clonal selective advantage tend to manifest late-onset and milder hematological features over at least 3 decades, thus representing a powerful rationale of the possible gene therapy (GT) success in FA (Ramírez et al., 2021; Gregory et al., 2001).

Nevertheless, neither somatic mosaicism should be erroneously regarded as an unequivocal index to foretell a favorable prognosis nor reversion be regarded as a definite protection against the risk of solid tumors.

Mosaic subjects could indeed exhibit clinical features ranging from no evident FA signs to severe hematologic failure, regardless of the presence of a population of reverted clones. The explanation behind these observations entails that long-term hematological stability generally derives from reversion events in HSPCs, yet involving less than 1/6 of all FA mosaic cases (Fargo et al., 2014; Castella et al., 2011). Only a proper assessment of the origin of clonality and its extent beyond the lymphoid compartment will thus be informative of patients’ actual proclivity toward BMF (Hughes and Kurre, 2022).

Moreover, incertitude about the susceptibility of FA mosaic individuals to myeloid malignancies persists. Despite the presence of native mutant cells, these patients are commonly less prone to cancer development by virtue of the proliferative advantage of the corrected clones (Gregory et al., 2001; Tsai and Lindsley, 2020). In the case of the accumulation of cancer-driven mutations before the reversion event, it could be the very reverted population that exposes individuals to the tumoral onset instead (Tsai and Lindsley, 2020).

Eventually, since the dawn of FA therapy, hematopoietic stem cell transplantation (HSCT) has remained the only curative approach for hematological defects, performed in the case of marrow function below the level of transfusion dependence or blood malignant evolution (Dufour, 2017; Fanconi Anemia Research Fund, 2022). This procedure, however, could still imply important life-threating consequences (e.g., graft-versus-host disease and increased solid tumor risks) (Kutler et al., 2003; Ramírez et al., 2021; Fargo et al., 2014; Kalb et al., 2007), from which FA mosaic patients could be spared or exposed at later ages by means of a more attentive evaluation of their own reversion-triggering events. Conversely, whether HSCT would be necessary, the chimeric state of FA and corrected cells in mosaic patients could hamper the immunosuppression provided by the standard low-conditioning regimen used in FA therapeutics (MacMillan et al., 2000).

To sum up, herein, we illustrated the case of II-2, an FA proband with somatic mosaicism likely due to a gene conversion event and associated with a moderate clinical presentation. Our findings highlight the relevance to investigate the positive role of “disease modifier events” for a clearer prognosis interpretation and new personalized therapeutic strategies, enabling more accurate treatments and decision plans for the sizable subset of FA mosaic individuals and, by means, patients undergoing GT. The molecular diagnosis plays an important role in defining the proper surveillance for the “extra hematological complications” of FA, including screening for head and neck tumors, skin cancers, and endocrine dysfunctions.

## Data Availability

The original contributions presented in the study are publicly available. This data can be found here: https://www.ncbi.nlm.nih.gov/bioproject/PRJNA1013760.
